# Predictors and outcomes of successful primary nasal intermittent positive pressure ventilation in extremely preterm infants: A retrospective observational study

**DOI:** 10.5339/qmj.2025.94

**Published:** 2025-09-08

**Authors:** Ratheesh Paramban, Jojo Furigay, Sabry Nasr, Jihad Al Shouli, Ashraf Gad

**Affiliations:** 1 Department of Critical Care, Neonatal Intensive Care Unit, Women’s Wellness and Research Center, Hamad Medical Corporation, Doha, Qatar *Email: agad2@hamad.qa

**Keywords:** Extremely preterm, respiratory distress syndrome, surfactant, nasal intermittent positive pressure ventilation, neonates, continuous positive airway pressure

## Abstract

**Objectives:**

This study aimed to evaluate the effectiveness and short-term outcomes of primary nasal intermittent positive pressure ventilation (NIPPV) in extremely preterm (EP) infants with respiratory distress syndrome (RDS).

**Methods:**

A retrospective case–control study was conducted at the Women’s Wellness and Research Center in Qatar, from January 2017 to December 2019. Primary NIPPV success was defined as the absence of surfactant administration or mechanical ventilation within the first 72 hours of life.

**Results:**

Of 367 infants requiring respiratory support at birth, 69 were managed with primary NIPPV. Among them, 62.3% succeeded (NIPPV-S) and 37.7% failed (NIPPV-F). Birth weights (956 g vs. 937 g) and gestational ages (26.3 weeks vs. 26.2 weeks) were similar between groups. NIPPV-S babies had more vaginal deliveries (48.8% vs. 19.2%, *p* = 0.014), higher arterial pH levels (7.36 vs. 7.29, *p* < 0.001), lower initial FiO_2_ (27.8% vs. 35.3%, *p* < 0.001), and less severe RDS (2.5% vs. 28.6%, *p* = 0.006). They also received caffeine earlier (2.6 hours vs. 4.3 hours, *p* = 0.042) and were more often in room air at day 28 (34.9% vs. 8.2%, *p* = 0.016). In the NIPPV-F group, 65% were intubated within 12 hours. NIPPV-S infants also had lower rates of brain injury (14.6% vs. 45.8%, *p* = 0.006) and bronchopulmonary dysplasia (BPD) (18.6% vs. 41.7%, *p* = 0.041), with a trend towards reduced death or severe BPD (2.3% vs. 15.4%, *p* = 0.063). Multivariate analysis identified admission FiO_2_ less than 0.4, vaginal delivery, and normal fetal Doppler as significant predictors of NIPPV success.

**Conclusions:**

Among EP infants managed with primary NIPPV, success within the first 72 hours was associated with lower initial FiO_2_, vaginal delivery, and normal fetal Doppler findings. EP infants who succeeded on NIPPV had less severe RDS and better outcomes, including lower rates of brain injury and BPD. Early identification of infants likely to succeed may improve clinical outcomes.

## 1. INTRODUCTION

Respiratory distress syndrome (RDS) is a leading cause of morbidity and mortality in preterm infants, particularly in extremely preterm (EP) infants (less than 28 weeks of gestation). Unlike older moderate preterm (32–33 weeks of gestation) or late preterm infants (34–36 weeks of gestation), EP infants typically require prolonged ventilatory support and an extended stay in the neonatal intensive care unit (NICU). This disease is characterized by insufficient surfactant production and the structural immaturity of the lungs.^1,2^ Over the years, the ventilatory management of RDS has evolved, with continuous positive airway pressure (CPAP) therapy and mechanical ventilation (MV) serving as traditional stalwarts of treatment.^3^ However, these interventions are not without risks. MV is associated with an increased risk of bronchopulmonary dysplasia (BPD), while CPAP alone may not be sufficient for the most vulnerable infants.^3–5^

Nasal intermittent positive pressure ventilation (NIPPV) is a form of non-invasive ventilation (NIV) that has emerged as an alternative therapy, combining the benefits of CPAP with those of MV. This method potentially reduces the need for intubation by providing additional breaths that support the infant’s own respiratory efforts.^4^ NIPPV is believed to improve gas exchange and improve lung recrutment, leading to better respiratory outcomes for EP infants.^6^ Despite its increasing use, the evidence for the effectiveness of NIPPV and the identification of factors predictive of its success or failure in this population remain areas of active investigation.^5,7–10^

In the realm of neonatal care, there is a burgeoning recognition of the need for less invasive respiratory support techniques. A systematic review by Ramaswamy et al. suggested that NIPPV reduces the incidence of BPD and the need for subsequent intubation compared to CPAP alone.^6^ A recent meta-analysis including 17 trials with nearly 2,000 preterm infants revealed that early NIPPV is superior to CPAP in reducing the risk of respiratory failure and the need for intubation and ventilation in very preterm infants with RDS. Furthermore, the analysis indicated a possible reduction in the rate of BPD with NIPPV.^11^ However, data from most studies lacked specific focus on EP infants, highlighting the need for additional research in this high-risk population.

In our practice, many EP infants are started on NIV, particularly those weighing less than 800 grams at birth, receive NIPPV as primary respiratory support. Other spontaneously breathing infants are usually managed with CPAP, often with a positive end expiratory pressure (PEEP) of 6–8 cmH_2_O.

To our knowledge, no studies have specifically evaluated the effectiveness of primary NIPPV in EP infants. The study aims to address this gap by assessing a cohort of EP infants managed with NIPPV as their initial respiratory support. The objectives were to identify predictors of successful treatment and short-term outcomes. The results of this study may provide valuable insights, support clinical decision-making, and contribute to the development of evidence-based guidelines for managing RDS in EP infants.

## 2. MATERIALS AND METHODS

### 2.1. Design, population, study setting/location

This single-center, retrospective observational study was conducted at the Women’s Wellness and Research Center (WWRC), previously known as Women’s Hospital, in Doha, Qatar. The study obtained ethical approval from the WWRC Medical Research Center (MRC)/Institutional Review Board (IRB) under protocol number MRC-01-20-1114. As the largest tertiary care center in the country, WWRC recorded a total of 51,625 deliveries during the study period from January 2017 to December 2019. Among these deliveries, 367 EP infants were admitted to the level III NICU, excluding those periviable newborns who did not receive delivery room resuscitation. The study focused on EP infants who were managed with NIPPV as the primary mode of respiratory support immediately after birth.

### 2.2. Inclusion and exclusion criteria

All live-born EP infants with a gestational age (GA) of <28 weeks, who were admitted to the NICU and received NIPPV as the primary mode of respiratory support during the study period, were included in the analysis. EP infants initially managed with CPAP and then switched to NIPPV in the delivery room, as indicated by the treating physician within the first hour, were considered to have received primary NIPPV therapy. Exclusion criteria included infants who were intubated in the delivery room, those who were placed on other forms of noninvasive respiratory support beyond one hour after birth, infants who died within 12 hours of age, and those with multiple congenital anomalies.

### 2.3. Objectives

The main objectives of this study were to identify factors contributing to NIPPV success (NIPPV-S) and failure (NIPPV-F) as a primary mode of respiratory support in EP infants, and to compare various clinical outcomes between the two groups. The success of primary NIPPV was defined as the continuation of NIPPV support beyond a 72-hour period or successful weaning to CPAP, nasal cannula, or complete discontinuation of the mode, without the need for intubation, mechanical ventilation, or surfactant therapy.

### 2.4. Data collection

Data were collected from maternal and neonatal charts. The maternal dataset included maternal age, antenatal steroid doses, diabetes, hypertension, premature rupture of membrane, chorioamnionitis, group B streptococcal colonization, antenatal ultrasound, oligohydramnios, umbilical artery (UA) Doppler, fetal distress, and the mode of delivery. The neonatal dataset included GA, birth weight (BW), resuscitation at birth, positive pressure ventilation (PPV), respiratory severity score (RSS), calculated as the product of mean airway pressure (MAP) multiplied by the fraction of inspired oxygen (FiO_2_), cord gas pH, and NICU respiratory characteristics such as respiratory support in the NICU, maximum NIPPV pressure and PEEP, maximum FiO_2_ in the NICU, blood gas, time of intubation and number of surfactant doses, and chest X-ray findings of RDS severity as mild (ground-glass appearance without significant air bronchograms), moderate (more pronounced ground-glass opacities with visible air bronchograms), and severe (diffuse ground-glass opacities “white-out” and prominent air bronchograms).^12^ Furthermore, clinical characteristics were recorded, such as work of breathing (WOB), pulmonary hypertension, sepsis, ventilator-associated pneumonia (VAP), pneumothorax, necrotizing enterocolitis (NEC), patent ductus arteriosus (PDA), retinopathy of prematurity (ROP), hypotension, blood transfusion, and oxygen requirements at 28 days and corrected age of 36 weeks. WOB was assessed based on the Silverman–Andersen score of mild (0–3), moderate (4–7), and severe (8–10) RDS.^13^

### 2.5. Statistical analysis

Descriptive analyses were conducted to evaluate patient characteristics and clinical variables. Chi-square or Fisher’s exact test was used for binary variables to analyze differences between groups (NIPPV-S vs. NIPPV-F). The distribution of the data was assessed using the Shapiro–Wilk test. Continuous variables were analyzed using the Student’s t-test if the data followed a normal distribution. Mean and standard deviation (SD) were used to present continuous variables, while numbers and percentages were used for categorical variables. A stepwise logistic regression (backward LR) was conducted to examine the associations between various exposures and NIPPV-S at different time points after controlling for potential confounders. The multivariable model included all significant variables associated with successful NIPPV identified in the univariate analyses, with a significance level of 0.1. All *p* values were two-tailed, and values below 0.05 were considered statistically significant. Statistical analyses were performed using SPSS for Windows (version 29.0, IBM Corp., Armonk, NY, USA).

## 3. RESULTS

**Figure 1.**
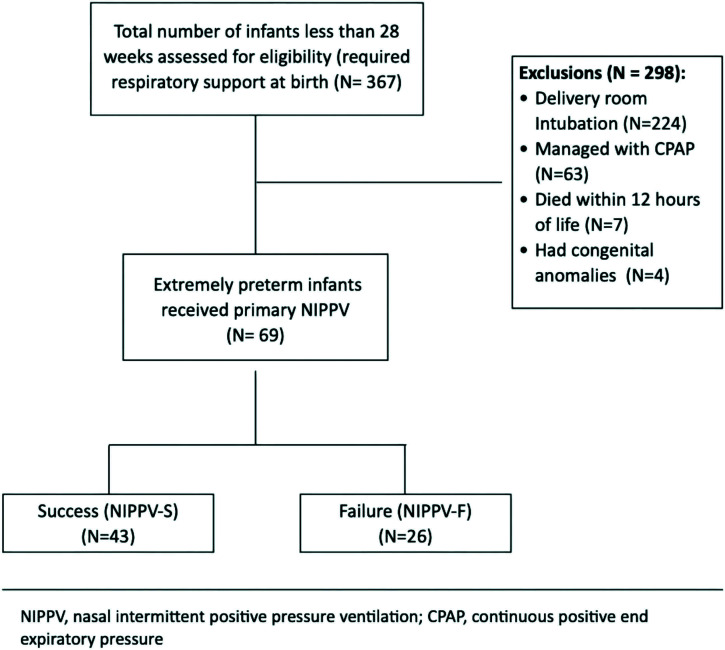
Flow chart illustrating the study selection process for nasal intermittent positive pressure ventilation.

During the study period, a total of 367 EP infants were born. Among these, 11 infants were excluded from the study for various reasons (seven deaths and four infants with congenital anomalies). Among the remaining infants, 224 were intubated in the delivery room, while 63 were placed on CPAP therapy and continued for more than 1 hour of life. The final analysis included 69 infants managed with NIPPV as primary respiratory support, consisting of 43 infants (62.3%) in the NIPPV success (NIPPV-S) group and 26 (37.7%) in the NIPPV failure (NIPPV-F) group (Figure 1).

Table 1 describes the maternal and neonatal demographics of the study cohort. Most patients were Middle Eastern(57.9%), while Asians and Caucasians represented 30.4% and 11.6% of the cases, respectively. Over 95% of mothers received antenatal steroids; however, only 71% received two doses, with higher rates noted in NIPPV-S cases (*p* = 0.062). Neonates in the NIPPV-S group had lower rates of abnormal fetal Doppler (4.9% vs. 17.9%), with an odds ratio (OR) of 0.205 and a 95% confidence interval (CI) of 0.037–1.147, although this was not statistically significant (*p* = 0.095). Among the seven patients identified with abnormal fetal Doppler findings, three had reversed end-diastolic flow (EDF) in the ductus venosus and umbilical artery (UA), along with high resistance indices and absent EDF. The remaining four patients had elevated resistance indices without absent or reversed EDF.

The two groups had similar BW and GA characteristics, with a mean GA of 26.3 weeks and a mean BW of 956 grams. The cohort included 38 females and 31 males. Less infants in the NIPPV-S group were delivered via cesarean section (CS) (51.2% vs. 80.8% in the NIPPV-F group), OR 0.249; 95% CI 0.079–0.783, *p* = 0.014. No other significant differences in baseline maternal and neonatal characteristics or delivery circumstances were observed.

Delivery room management of both groups, including PPV, ventilation parameters, and cord gas values, was analyzed as detailed in Table 2. The two groups had similar characteristics and requirements after birth, with no differences in baseline RSS and WOB before the initiation of NIPPV. Although most infants had breathing efforts at birth, one-third still received PPV delivered by nasal mask, and 65.2% received a PEEP of approximately 6 cmH_2_O. No other significant differences were noted between the groups. The mean FiO_2_ was greater than 0.3 in both groups before applying NIPPV.

After applying NIPPV, the NIPPV-S group had lower FiO_2_ requirements compared to the NIPPV-F group (28.3 ± 6.2 vs. 34.2 ± 14.5; mean difference (MD) 5.854; 95% CI 0.898–10.788, *p* = 0.021). Furthermore, infants in the NIPPV-S group tended to have higher cord arterial pH and lower base deficits compared to the NIPPV-F group (2.3% vs. 15.4%), although this difference was not statistically significant (*p* = 0.063). NIPPV PEEP was lower in the success group (5.9 ± 0.4 vs. 6.2 ± 0.5; MD 0.262; 95% CI 0.045 – 0.479, *p* = 0.009); however, the MAP set did not differ between the two groups.

Table 3 presents a detailed comparison of the clinical and respiratory management parameters between the NIPPV-S and NIPPV-F groups in the NICU. Infants in the NIPPV-S group had significantly higher admission arterial pH levels compared to those in the NIPPV-F group (7.36 ± 0.6 vs. 7.29 ± 0.7, *p* < 0.001) and lower paCO_2_ levels (41.9 ± 7.1 mmHg vs. 47.4 ± 10.2 mmHg, *p* = 0.009). During the first 24 hours in the NICU, infants in the NIPPV-S group required lower FiO_2_ (0.28 ± 0.054 vs. 0.35 ± 0.09; MD 7.509; 95% CI 4.121–10.897, *p* < 0.001) before escalation in the NIPPV-F group. Similarly, the RSS was lower in NIPPV-S patients (3.44 ± 0.9 vs. 4.15 ± 1.3; MD 0.715; 95% CI 0.1779–1.252, *p* = 0.010). Additionally, the initial X-ray showed less severe RDS in the NIPPV-S group (2.5% vs. 28.6%, OR 0.046; 95% CI 0.005–0.419, *p* = 0.006).

Caffeine was administered earlier to the NIPPV-S group than to the NIPPV-F group (2.64 ± 1.44 hours vs. 4.33 ± 5.00 hours, MD 1.69; 95% CI 0.060–3.327, *p* = 0.042). In the NIPPV-S group, only three infants (7%) were later intubated due to hypoactivity, increased FiO_2_ requirements, or increased WOB; all of these patients developed culture-proven late-onset sepsis, none received surfactant therapy, and required mechanical ventilation for 2.3 ± 1.5 days. Furthermore, the timing of intubation in the NIPPV-S group was significantly delayed (181 ± 92 hours) compared to the NIPPV-F group (9.4 ± 8.5 hours; MD −172; 95% CI −206.2 to −13.7 hours, *p* < 0.001). In the NIPPV-F group, 65% of the patients were intubated within 12 hours and 35% within 24 hours. All patients in the latter group received surfactant: 19 patients (73.1%) received one dose, and seven patients (26.9%) received two or more doses. Surfactant was administered using the modified intubation–surfactant–extubation (INSURE) technique in 46.1% of the NIPPV-F group (extubated within 24 hours), and the remaining 53.9% of cases received mechanical ventilation for 7.6 ± 6.7 days.

The duration of primary NIPPV therapy was significantly longer in the NIPPV-S group compared to the NIPPV-F group (36.0 ± 28 hours vs. 9.8 ± 21 hours, MD −26.283; 95% CI −38.897 to −13.667, *p* < 0.001). A higher percentage of infants in the NIPPV-S group were in room air by 28 days of life compared to those in the NIPPV-F group (34.9% vs. 8.2%). Conversely, more infants in the NIPPV-F group were still on NIPPV at 28 days (29.2% vs. 14.0%, *p* = 0.020). There were no significant differences observed in the rates of other NICU complications such as pneumothorax, blood transfusion, use of inhaled nitric oxide, vasopressors, or postnatal dexamethasone therapy.

Table 4 presents the results of the LR analysis that identified significant perinatal variables and clinical characteristics associated with the success of primary NIPPV in EP infants. After adjusting for several significant variables identified in the univariate analysis, it was found that FiO_2_ requirement levels ≤ 0.4 upon NICU admission were significantly associated with successful NIPPV outcomes (adjusted OR 17.790; 95% CI 0.3.818–82.892, *p* < 0.001). Additionally, vaginal delivery as opposed to CS correlated with NIPPV-S (aOR 4.184; 95% CI q.050–16.667, *p* = 0.042), and normal fetal Doppler findings were similarly correlated with increased odds of NIPPV-S (aOR 9.346; 95% CI 1.326–65.870, *p* = 0.025).

Variables recorded in step 1 of the backward stepwise LR include the following: abnormal fetal Doppler, cesarean delivery, maximum FiO_2_ before NIPPV, cord gas <7.2 or base deficit >−8, NICU gas pH <7.3, NICU paCO_2_ <50 mmHg, NICU base excess, FiO_2_ on NIPPV, NICU FiO_2_ ≤0.4, NIPPV PEEP, caffeine hour, two or more doses of antenatal steroids, NICU RSS on NIPPV, and NIPPV MAP.

Table 5 provides a comprehensive comparison of clinical outcomes between infants who succeeded NIPPV and those who failed. The NIPPV-S group exhibited significantly lower rates of intraventricular hemorrhage (IVH) (14.6% vs. 41.7%, OR 0.240; 95% CI 0.073–0.786, *p* = 0.015) and brain injury, including any IVH or periventricular leukomalacia (PVL) (14.6% vs. 45.8%, OR 0.203; 95% CI 0.062–0.660, *p* = 0.006). The incidence of BPD was also reduced in the NIPPV-S group (18.6% vs. 41.7%, OR 0.320; 95% CI 0.105–0.978, *p* = 0.041). Additionally, the combined rate of mortality or severe BPD was lower in the NIPPV-S group (2.3% vs. 15.4%, OR: 0.131; 95% CI 0.014–1.244, *p* = 0.063). There were no significant differences between the groups regarding rates of death, late-onset sepsis, VAP, PDA, ROP, NEC, or bowel perforation. The mean length of hospital stay was similar between the groups (72.4 ± 41.0 days for NIPPV-S vs. 80.3 ± 40.7 days for NIPPV-F, MD 7.95; 95% CI −12.328–28.239, *p* = 0.436).

## 4. DISCUSSION

This retrospective study evaluated the factors associated with the success of primary NIPPV support for EP infants with RDS. The majority of infants who received primary NIPPV achieved successful outcomes. The baseline characteristics and transition parameters were similar between the two groups, except for a lower rates of vaginal deliveries observed in the failure group. Infants in the NIPPV-S group demonstrated better respiratory parameters, including higher arterial pH levels, lower initial FiO_2_ requirements, milder RDS severity on X-ray, and improved outcomes with lower rates of brain injury and BPD. Key predictors for NIPPV-S included lower initial FiO_2_, vaginal delivery, and normal fetal Doppler upon NICU admission. These findings highlight the importance of early identification of these variables to guide interventions and improve NIPPV success in these babies.

Several studies demonstrated the effectiveness of NIPPV in reducing extubation failure, including the need for reintubation within 48 hours to one week, more effectively than nasal CPAP. However, its effect on BPD and mortality rates remains uncertain.^14,15^ This uncertainty was highlighted in a meta-analysis conducted by Kirpalani et al., which included more than a thousand extremely low BW infants during their first 28 days of life when NIV support was first implemented. The results of this meta-analysis did not reveal any significant benefit of NIPPV over CPAP in terms of mortality or BPD reduction, duration of respiratory support, or other clinically significant outcomes such as air leaks and NEC.^16^

Additionally, several studies have evaluated the efficacy of NIPPV as the primary respiratory support for preterm infants, showing a reduction in the need for intubation.^17–20^ However, some studies did not observe the same effect.^21,22^ In a systematic review and network meta-analysis conducted by Ramaswamy et al., four different modes of early respiratory support were reviewed in over 400 preterm infants with RDS across 35 studies. Their findings indicated that NIPPV was more effective in decreasing the requirement for MV compared to CPAP, with fewer air leaks and a lower incidence of BPD or mortality.^6^

In a recent meta-analysis conducted by Lemyre et al., which analyzed 17 studies comparing NIPPV and continuous CPAP initiated within the first 6 hours of life, it was concluded that infants supported with NIPPV had a lower incidence of respiratory failure and intubation.^11^ Moreover, respiratory support provided after birth with NIPPV using RAM cannula showed a significant decrease in the need for intubation (31% vs. 85%) in EP infants (24–27 weeks of gestation).^23^

Our study included a cohort of EP infants who received only NIPPV as the primary mode of respiratory support. Despite its distinct design when comparing early NIPPV to other forms of NIV, previous research has shown that lower GA and BW are significant risk factors contributing to the failure of early NIV use in early preterm infants.^24–27^ Similarly, a retrospective study conducted by Buyuktiryaki, which compared three NIV strategies (CPAP, nasal BiPAP, and NIPPV), found that the CPAP group experienced a higher failure rate compared to the other groups, with antenatal steroids and GA being significantly associated with these failures.^28^ However, our study found that infants in both groups who started on NIPPV exhibited similar basic characteristics, including maternal and neonatal demographics such as BW and GA.

The majority of infants in our study cohort received antenatal steroids; however, those in the success group received a full course of treatment more frequently, although the difference was not statistically significant. The study conducted by Yazici et al. demonstrated that the administration of antenatal steroids and NIPPV significantly improved overall success rates of NIV.^25^

Both groups in our study started with similar RSS and FiO_2_ requirements. However, after applying NIPPV, the NIPPV-S group exhibited better RSS scores, lower NIPPV PEEP, and lower FiO_2_ requirements. High FiO_2_ requirements in the delivery room after NIPPV stabilization and upon NICU admission predicted NIPPV-F, indicating more severe RDS, which was later confirmed by X-ray data. This finding is supported by a similar study on NIV failure in early preterm infants, which found that higher FiO_2_ during resuscitation and after surfactant administration were independent risk factors for NIV failure.^29^ Additionally, another study involving preterm infants (28–36 weeks of gestation) receiving primary NIV found that lower GA and higher FiO_2_ predicted the need for intubation within 72 hours.^30^

Similar to our study, NIV failure is often managed with intubation and surfactant administration.^29,31^ Although the NIPPV-S group received caffeine earlier, it did not emerge as an independent variable for NIPPV-S in the regression analysis, a larger sample may be needed to detect a potential significant effect. Earlier caffeine therapy has been shown to enhance the effectiveness of NIPPV and improve BPD outcomes.^32–34^.

Our results suggest that fewer infants in the success group were delivered via CS, indicating less severe respiratory distress, making CS delivery an independent predictor of NIPPV-F. Infants delivered via CS, especially without labor, often experience delayed clearance of lung fluid compared to those delivered vaginally, exacerbating respiratory distress and making NIV less effective. Additionally, deliveries via CS lack labor-associated hormonal changes, particularly the surge in catecholamines, which can reduce surfactant production and release. CS deliveries are often performed due to complications such as pre-eclampsia and fetal distress. The association between CS delivery and the exacerbation of respiratory distress is supported by several studies and meta-analyses.^35–37^. A meta-analysis of 26 studies found that the pooled OR for the risk of neonatal RDS was 2.38 (95% CI 1.89–2.99) for elective CS and 1.85 (95% CI 1.34–2.56) for emergency CS.^37^

An important difference between the NIPPV-S and NIPPV-F groups is the higher pCO_2_ levels and lower pH values in the failed group. Respiratory and significant metabolic acidosis are usually associated with unfavorable respiratory outcomes and may indicate inadequate respiratory support due to poor alveolar ventilation.^11^ Additionally, abnormal fetal Doppler emerged as another independent predictor for NIPPV-S in our study. Abnormal fetal Doppler findings, such as absent or reversed EDF in the UA and abnormal main pulmonary artery indices, may indicate placental insufficiency, fetal hypoxia, and lung disease. Consistent with findings from previous studies, these conditions may impair lung development, leading to structural abnormalities and reduced surfactant production, ultimately leading to worsened respiratory outcomes.^38–40^ However, due to the small sample size in our study, this finding should be validated through larger prospective studies. Moreover, lower GA and low Apgar scores were considered to be independent predictors of NIV failure.^27,41^

Our findings demonstrated several significant differences in short-term outcomes between the NIPPV-S and NIPPV-F groups. Infants in the NIPPV-S group showed lower rates of complications. For example, the incidence of brain injury (including any IVH or PVL) was significantly lower in the NIPPV-S group compared to the NIPPV-F group. Similarly, the incidence of BPD was lower in the success group. This finding aligns with previous studies on NIV success.^29,31,42^ The association between NIPPV-S and reduced BPD and brain injury in EP infants could be attributed to both patient- and NIPPV-related factors. Infants with less RDS and better baseline clinical status are more likely to experience benefits.

Our study addressed a critical gap in neonatal research by exclusively evaluating primary NIPPV treatment in EP patients with RDS. The study included detailed clinical variables, allowing for extensive analysis. Its practical implications were significant because it identified limitations in the study, including its retrospective design, which may have introduced biases due to potentially incomplete or inaccurate medical records, complicating the establishment of a causal relationship. Additionally, the study was limited to a single center, which raises concerns regarding the generalizability of the findings to other settings or populations. While CPAP was initially used for all infants before transitioning to NIPPV, we did not analyze the specific clinical factors that influenced this transition. Although both groups had similar baseline characteristics and initial respiratory support settings, the decision to switch to NIPPV was determined by clinicians, which may introduce some selection bias. However, given the comparable PEEP levels and the timing of NIPPV initiation, we believe that this had a minimal impact on our findings. Future studies using a standardized protocol for transitioning from CPAP to NIPPV could further elucidate this aspect. The small sample size in our study may have reduced statistical power and the ability to detect other predictors of NIPPV-S. The definition of NIPPV-S used in this study, which is characterized by the absence of surfactant administration or the need for intubation within the first 72 hours, may not fully capture clinical success.

## 5. CONCLUSION

This study demonstrates that primary NIPPV can support many EP infants with RDS without the need for mechanical ventilation or surfactant administration within the first 72 hours. Key predictors of NIPPV-S included lower initial FiO_2_ requirements, vaginal delivery, and normal fetal Doppler ultrasound results. These findings can provide guidance for early identification of infants who are likely to benefit from NIPPV, thereby optimizing respiratory management strategies and potentially mitigating brain injury and BPD. As the findings suggest, most infants started on NIPPV were successfully managed, indicating that NIPPV may be an effective primarily mode for providing respiratory support in EP infants with RDS. These results highlight the need for individualized respiratory support strategies in this population. Further prospective studies with large sample sizes are essential to validate these findings and to address the limitations present in our study.

## LIST OF ABBREVIATIONS

**Table:** 

ANS	Antenatal Steroids
PD	Bronchopulmonary Dysplasia
BW	Birth Weight
CI	Confidence Interval
CPAP	Continuous Positive Airway Pressure
CS	Cesarean Section
EDF	End-Diastolic Flow
EP	Extremely Preterm
FiO_2_	Fraction of Inspired Oxygen
GA	Gestational Age
GBS	Group B Streptococcus
INSURE	Intubation–Surfactant–Extubation
IVH	Intraventricular Hemorrhage
MAP	Mean Airway Pressure
MD	Mean Difference
MV	Mechanical Ventilation
NC	Nasal Cannula
NEC	Necrotizing Enterocolitis
NICU	Neonatal Intensive Care Unit
NIPPV	Nasal Intermittent Positive Pressure Ventilation
NIV	Non-Invasive Ventilation
OR	Odds Ratio
PDA	Patent Ductus Arteriosus
PEEP	Positive End-Expiratory Pressure
PPHN	Persistent Pulmonary Hypertension
PPROM	Preterm Premature Rupture of Membranes
PPV	Positive Pressure Ventilation
PVL	Periventricular Leukomalacia
RDS	Respiratory Distress Syndrome
ROP	Retinopathy of Prematurity
RSS	Respiratory Severity Score
SD	Standard Deviation
SIP	Spontaneous Intestinal Perforation
UA	Umbilical Artery
UTI	Urinary Tract Infection
VAP	Ventilator-Associated Pneumonia
WOB	Work of Breathing
WWRC	Women’s Wellness and Research Center

## ETHICAL STATEMENT

The study was conducted in complete adherence to the principles of the Declaration of Helsinki, Good Clinical Practice (GCP), and in accordance with the laws and regulations of the Ministry of Public Health in Qatar. Ethical approval for the study was granted by the WWRC Medical Research Center (MRC)/Institutional Review Board (IRB) under protocol number MRC-01-20-1114.

## AUTHORS’ CONTRIBUTION

AG conceptualized and designed the study and performed the statistical analysis. RP and AG drafted the study protocol, supervised the data collection, and drafted the first manuscript. RP, JF, SN, and JA collected the data for the study. All authors read and approved the final version of the manuscript.

## ACKNOWLEDGMENTS

The authors express their special thanks to the entire NICU and respiratory therapy staff at WWRC for providing high-quality care to newborns.

## CONFLICT OF INTEREST

The authors have no conflicts of interest to declare.

## Figures and Tables

**Table 1.** Maternal and neonatal characteristics of extremely preterm infants who received nasal intermittent positive pressure ventilation at birth.

	NIPPV-success (*n* = 43)	NIPPV-failure (*n* = 26)	OR (95% CI)/mean difference (95% CI)	*P*
**Maternal**				
Maternal age (years), mean ± SD	29.2 ± 5.6	29.4 ± 5.3	0.992 (0.887–1.110)	0.891
Ethnicity, *n* (%)				
Caucasian	5 (11.6%)	3 (11.5%)	1.0	
Middle Eastern	26 (60.5%)	14 (53.8%)	1.114 (0.231–5.366)	0.893
Asian	12 (27.9%)	9 (34.6%)	0.800 (0.150–4.258)	0.794
Antenatal steroids (ANS), *n* (%)	42 (97.7%)	24 (92.3%)	3.500 (0.301–40.652)	0.552
ANS doses ≥2, *n* (%)	34 (79.1%)	15 (57.7%)	2.770 (0.950–8.078)	0.062
Timing of ANS before delivery (days), mean ± SD	6.62 ± 7.3	9.2 ± 8.6	2.555 (−1.479–6.589)	0.210
Antenatal magnesium sulfate, *n* (%)	3 (7.3%)	4 (15.4%)	0.413 (0.085–2.012)	0.413
Hypertension, *n* (%)	5 (11.6%)	6 (23.1%)	0.439 (0.0119–1.617)	0.309
Gestational diabetes, *n* (%)	10 (23.3%)	7 (26.9%)	0.823 (0.269–2518)	0.732
GBS, *n* (%)	6 (14.3%)	3 (13.0%)	1.111 (0.250–4.929)	1.000
UTI, *n* (%)	6 (14%)	2 (7.7%)	1.946 (0.362–10.449)	0.701
Fetal distress, *n* (%)	2 (4.7%)	3 (11.5%)	0.374 (0.058–2.404)	0.359
Chorioamnionitis, *n* (%)	1 (2.3%)	3 (11.5%)	0.183 (0.018–1.857)	0.147
PPROM, *n* (%)	5 (14.7%)	2 (8.7%)	1.810 (0.320–10.246)	0.689
Oligohydramnios, *n* (%)	3 (75%)	3 (75%)	1.000 (0.041–24.547)	1.000
PPROM duration (hours), mean ± SD	6.7 ± 17.1	8.7 ± 30.0	1.960 (−10.564–14.485)	0.755
Amniotic fluid index, mean ± SD	6.8 ± 4.2	6.2 ± 3.5	−0.7116 (−2.699–1.276)	0.477
Abnormal fetal Doppler, n (%)	2 (4.9%)	5 (17.9%)	0.205 (0.037–1.147)	0.095
General anesthesia, *n* (%)	1 (2.4%)	3 (11.5%)	0.187 (0.018–1.903)	0.152
**Neonatal**				
Birth weight (grams), mean ± SD	968 ± 134	937 ± 165	−31.472 (−103.962–41.017)	0.389
Gestational age (weeks), mean ± SD	26.4 ± 0.7	26.2 ± 0.9	−0.218 (−0.617–0.181)	0.279
SGA, *n* (%)	7 (16.3%)	6 (23.1%)	0.648 (0.191–2.195)	0.480
Female gender, *n* (%)	23 (53.5%)	15 (57.7%)	0.843 (0.316–2.252)	0.734
Multiple birth, *n* (%)	16 (37.2%)	8 (30.8%)	1.333 (0.473–3.762)	0.580
Cesarean delivery, *n* (%)	20 (51.2%)	21 (80.8%)	0.249 (0.079–0.783)	0.014
One-minute Apgar <7, *n* (%)	14 (32.6%)	7 (26.9%)	1.310 (0.447–3.843)	0.622
Five-minute Apgar <7, *n* (%)	2 (4.65%)	1 (3.8%)	1.220 (0.105–14.152)	0.874

NIPPV: Nasal intermittent positive pressure ventilation, SD: Standard deviation, GBS: Group B streptococcus, UTI: Urinary tract infection, PPROM: preterm premature rupture of membranes, SGA: small for gestational age.

An OR of 1.0 indicates a reference value.

**Table 2.** Delivery room respiratory management of extremely preterm infants who received nasal intermittent positive pressure ventilation at birth.

	NIPPV-success (*n* = 43)	NIPPV-failure (*n* = 26)	OR (95% CI)/mean difference (95% CI)	*P*
Received PPV, n (%)	17 (39.5%)	8 (30.8%)	1.471 (0.524–4.134)	0.463
PPV duration (min), mean ± SD	1.46 ± 1.2	0.96 ± 0.70	−0.502 (−1.456–0.451)	0.287
RSS prior to NIPPV, mean ± SD	1.90 ± 0.41	1.98 ± 0.68	0.0838 (−0.179–0.346)	0.527
Breathing, *n* (%)	30 (68.8%)	22 (84.6%)	0.420 (0.120–1.462)	0.165
Work of breathing, *n* (%)				
Mild	12 (27.8%)	8 (30.8%)	1.0	
Moderate	30 (69.8%)	15 (57.7%)	1.333 (0.449–3.959)	0.604
Severe	1 (2.3%)	3 (11.5%)	0.420 (0.019–2.533)	0.226
CPAP PEEP prior to NIPPV (cmH_2_O), mean ± SD	5.72 ± 0.59	5.77 ± 0.58	0.048 (−0.244–0.341)	0.742
FiO_2_ prior to NIPPV (%), mean ± SD	0.33 ± 0.06	0.34 ± 0.09	0.899 (−2.575–4.373)	0.607
NIPPV start (min), mean ± SD	26.2 ± 12.6	23.8 ± 12.5	−2.316 (−8.541–3.907)	0.460
NIPPV PIP (cmH_2_O), mean ± SD	18.8 ± 2.8	18.7 ± 2.4	0.037 (−0.908–0.834)	0.933
NIPPV PIP (>20 cmH_2_O), *n* (%)	14 (32.6%)	12 (46.2%)	0.563 (0.207–1.532)	0.261
NIPPV PEEP (cmH_2_O), mean ± SD	5.9 ± 0.4	6.2 ± 0.5	0.262 (0.045–0.479)	0.009
NIPPV PEEP (>7 cmH_2_O), *n* (%)	2 (4.7%)	4 (16.7%)	0.268 (0.045–1.582)	0.146
NIPPV rate (RR/min), mean ± SD	40.0 ± 4.3	40.8 ± 3.9	0.769 (−1.316–2.854)	0.464
NIPPV rate (50–60 RR/min), *n* (%)	4 (9.3%)	2 (8.3%)	1.128 (0.191–6.663)	1.000
NIPPV Ti (sec), mean ± SD	0.54 ± 0.10	0.52 ± 0.10	−0.019 (−0.717–0.0336)	0.469
NIPPV Ti (0.55–1.0 sec), *n* (%)	11 (25.6%)	3 (11.5%)	2.635 (0.660–10.523)	0.170
NIPPV MAP (cmH_2_O), mean ± SD	12.3 ± 1.1	12.7 ± 0.6	0.394 (−0.618–0.849)	0.089
FiO_2 _(%) on NIPPV, mean ± SD	0.28 ± 0.06	0.34 ± 0.14	5.843 (0.898–10.788)	0.021
Cord arterial pH <7.2 or base excess >−8, *n* (%)	1 (2.3%)	4 (15.45%)	0.131 (0.014–1.244)	0.063

NIPPV: nasal intermittent positive pressure ventilation, PPV: positive pressure ventilation, SD: standard deviation, RSS: respiratory severity score, CPAP: continuous positive airway pressure, PEEP: positive end-expiratory pressure, FiO_2_: fractional inspiratory oxygen, PIP: peak inspiratory pressure, Ti: inspiratory time, MAP: mean airway pressure.

NIPPV was provided by a nasal mask interface in all cases.

An OR of 1.0 indicates a reference value.

**Table 3.** Characteristic and respiratory management of extremely preterm infants who received nasal intermittent positive pressure ventilation at birth upon admission to the neonatal intensive care unit.

	NIPPV-success (*n* = 43)	NIPPV-failure (*n* = 26)	OR (95% CI)/mean difference (95% CI)	*p*
pH, mean ± SD	7.36 ± 0.6	7.29 ± 0.7	−0.067 (−0.0991 – −0.0368)	<0.001
PaCO_2_ (mmHg), mean ± SD	41.9 ± 7.1	47.4 ± 0.2	5.571 (1.439–9.702)	0.009
Lactate (mmol/L), mean ± SD	1.64 ± 0.7	1.88 ± 1.1	0.256 (−0.1939–0.7061)	0.260
BE (mmol/L), mean ± SD	−1.53 ± 2.3	−2.55 ± 2.90	−1.013 (−2.270–0.243)	0.112
Hb (g/dL), mean ± SD	16.0 ± 2.1	15.2 ± 2.5	−0.784 (−1.893–0.325)	0.163
FiO_2 _(%) on NIPPV, mean ± SD	0.28 ± 0.054	0.35 ± 0.87	7.509 (4.121–10.897)	<0.001
Caffeine bolus timing (hour), mean ± SD	2.64 ± 1.44	4.33 ± 5.00	1.694 (0.060–3.327)	0.042
RSS on NIPPV, mean ± SD	3.44 ± 0.91	4.15 ± 1.31	0.715 (0.1779–1.252)	0.010
Early-onset sepsis/congenital pneumonia	1 (2.4%)	1 (4.2%)	0.585 (0.035–9.793)	1.000
RDS severity (X-ray), *n* (%)				
Mild	26 (65%)	11 (39.3%)	1.0	
Moderate	13 (32.5%)	9 (32.1%)	0.694 (0.226–2.136)	0.525
Severe	1 (2.5%)	8 (28.6%)	0.046 (0.005–0.419)	0.006
NIPPV duration (hours), mean ± SD	36.0 ± 27.6	9.8 ± 21.3	−26.283 (−38.897 – −13.667)	<0.001
Intubation timing (hours), mean ± SD	181.3 ± 92	9.4 ± 8.5	−171.8 (−206.238 – −137.416)	<0.001
Pneumothorax, *n* (%)	0 (0.0%)	1 (4.2%)	0.365 (0.265–0.506)	0.375
Blood transfusion, *n* (%)	2 (5.0%)	2 (8.0%)	0.590 (0.078–4.475)	0.620
PPHN, *n* (%)	1 (2.4%)	2 (8.0%)	0.274 (0.024–3.185)	0.282
Hypotension at 7 days, *n* (%)	0 (0.0%)	1 (4.2%)	0.365 (0.265–0.506)	0.375
Postnatal dexamethasone*, *n* (%)	1 (2.5%)	2 (9.1%)	0.256 (0.022–3.002)	0.285
Respiratory support at 28 days, *n* (%)				
Room air/low flow NC, *n* (%)	15 (34.9%)	2 (8.4%)	1.0	
CPAP, *n* (%)	22 (51.2%)	15 (62.4%)	0.196 (0.039–0.983)	0.048
Intermittent ventilation, *n* (%)	6 (14.0%)	7 (29.2%)	0.114 (0.018–0.716)	0.020

NIPPV: non-invasive positive pressure ventilation, BE: base excess, Hb: hemoglobin, FiO_2_: fractional inspiratory oxygen, RSS: respiratory severity score, RDS: respiratory distress syndrome, PPHN: persistent pulmonary hypertension, NC: nasal cannula, CPAP: continuous positive airway pressure, MV: mechanical ventilation.

*Missing data: postnatal dexamethasone (*n* = 7).

An OR of 1.0 indicates a reference value.

**Table 4.** Significant perinatal and clinical characteristics associated with primary intermittent positive pressure ventilation success in extremely preterm infants.

	Adjusted OR	95% CI of the OR	*p*
**NICU admission FiO**_2_**≤ 0.4** Yes No	17.790 1.0	3.818–82.890	<0.001
**Normal fetal Doppler** Yes No	9.346 1.0	1.326–65.870	0.025
**Vaginal delivery** Yes No	4.184 1.0	1.050–16.667	0.042

The reference value for vaginal birth is cesarean section.

NICU: Neonatal intensive care unit, FiO_2_: Fraction of inspired oxygen.

**Table 5.** Short-term outcomes of extremely preterm infants who received intermittent positive pressure ventilation after birth.

	NIPPV-success (*n* = 43)	NIPPV-failure (*n* = 26)	OR (95% CI)/mean difference (95% CI)	*p*
Late-onset sepsis, *n* (%)	11 (25.6%)	3 (12.0%)	2.521 (0.630–10.093)	0.182
VAP, *n* (%)	2 (4.8%)	2 (8.3%)	0.550 (0.072–4.179)	0.618
IVH, *n* (%)	6 (14.6%)	10 (41.7%)	0.240 (0.73–0.786)	0.015
Brain injury (IVH/PVL), *n* (%)	6 (14.6%)	11 (45.8%)	0.203 (0.062–0.660)	0.006
PVL, *n* (%)	0 (0%)	2 (8.0%)	0.371 (0.268–0.513)	0.149
ROP, *n* (%)	7 (17.1%)	9 (34.5%)	0.389 (0.124–1.224)	0.101
PDA, *n* (%)	12 (27.9%)	7 (26.9%)	1.051 (0.352–3.135)	0.929
SIP, *n* (%)	1 (2.3%)	1 (3.8%)	0.595 (0.036–9.943)	1.000
BPD, *n* (%)	8 (18.6%)	10 (41.7%)	0.320 (0.105–978)	0.041
BPD category, *n* (%)				
No	35 (81.4%)	14 (58.3%)	1.0	
Mild	2 (4.7%)	2 (8.3%)	0.400 (0.051–3.125)	0.382
Moderate	5 (11.6%)	7 (29.2%)	0.286 (0.078–1.053)	0.060
Severe	1 (2.3%)	1 (4.2%)	0.400 (0.023–6.848)	0.527
Death or severe BPD, *n* (%)	1 (2.3%)	4 (15.4%)	0.131 (0.014–1.244)	0.063
Death, *n* (%)	1 (2.3%)	3 (11.3%)	0.183 (0.018–1.857)	0.147
NEC, *n* (%)	5 (11.6%)	4 (15.4%)	0.724 (0.176–2.981)	0.720
Length of stay (days), mean ± SD	72.4 ± 41.0	80.3 ± 40.7	7.95 (−12.328–28.239)	0.436

NIPPV: Nasal intermittent positive pressure ventilation, VAP: Ventilator-associated pneumonia, IVH: Intraventricular hemorrhage, PVL: Periventricular leukomalacia, ROP: Retinopathy of prematurity, PDA: Patent ductus arteriosus, SIP: Spontaneous intestinal perforation, BPD: bronchopulmonary dysplasia, NEC: necrotizing enterocolitis.

Missing data: VAP (*n* = 3), IVH (*n* = 4), PVL (*n* = 5), brain injury (*n* = 4), ROP (*n* = 2), BPD (*n* = 2).
